# Associations between media parenting practices and early adolescent screen use

**DOI:** 10.1038/s41390-024-03243-y

**Published:** 2024-06-05

**Authors:** Jason M. Nagata, Angel Paul, Felicia Yen, Zacariah Smith-Russack, Iris Yuefan Shao, Abubakr A. A. Al-shoaibi, Kyle T. Ganson, Alexander Testa, Orsolya Kiss, Jinbo He, Fiona C. Baker

**Affiliations:** 1 Department of Pediatrics, University of California, San Francisco, San Francisco, CA USA; 2 Factor-Inwentash Faculty of Social Work, University of Toronto, Toronto, ON Canada; 3 Department of Management, Policy and Community Health, University of Texas Health Science Center at Houston, Houston, TX USA; 4 Center for Health Sciences, SRI International, Menlo Park, CA USA; 5 School of Humanities and Social Science, The Chinese University of Hong Kong, Shenzhen, Guangdong China; 6 School of Physiology, University of the Witwatersrand, Johannesburg, South Africa

## Abstract

**Background:**

To assess the prevalence of various media parenting practices and identify their associations with early adolescent screen time and problematic social media, video game, and mobile phone use.

**Methods:**

Cross-sectional data from Year 3 of the Adolescent Brain Cognitive Development (ABCD) Study (2019–2022) that included 10,048 adolescents (12–13 years, 48.3% female, 45.6% racial/ethnic minorities) in the US were analyzed using multiple linear regression analyses adjusting for potential confounders.

**Results:**

Parent screen use, family mealtime screen use, and bedroom screen use were associated with greater adolescent screen time and problematic social media, video game, and mobile phone use. Parental use of screens to control behavior (e.g., as a reward or punishment) was associated with higher screen time and greater problematic video game use. Parental monitoring of screens was associated with lower screen time and less problematic social media and mobile phone use. Parental limit setting of screens was associated with lower screen time and less problematic social media, video game, and mobile phone use.

**Discussion:**

Parent screen use, mealtime screen use, and bedroom screen use were associated with higher adolescent problematic screen use and could be limited in a family media use plan. Parental monitoring and limiting of screen time are associated with less problematic screen use.

**Impact statement:**

- Although the American Academy of Pediatrics provides guidance for screen use for children 5–18 years, there is a paucity of evidence-based guidance for media parenting practices, specifically for early adolescents.
- In a diverse sample of 10,048 early adolescents across the US, we found cross-sectional associations between parent, mealtime, and bedroom screen use and higher adolescent problematic screen use.
- Parental monitoring and limiting of adolescent screen time were cross-sectionally associated with less problematic screen use in our analytic sample and may be incorporated into a family media use plan.

## Introduction

In this era of technology and digital media, there has been growing concern surrounding the effects of screen use behaviors in children and adolescents. Examples of screen-based technology and digital media include, but are not limited to, social media, video games, and mobile phones. Social media consists of technologies and digital media that facilitate the creation, sharing, and exchange of information, ideas, and multimedia content.^1^ Social networks are a specific type of social media that focuses on building and connecting communities of people through profiles, and sharing content.^1^ Excessive screen use in children and adolescents has been linked to mental and physical health problems, obesity, sedentary behaviors, and sleep difficulties.^2–5^ As parents play key roles during childhood and adolescence, child-parent relationships, parenting styles, and home environments have been identified as factors that may contribute to children’s screen use patterns.^6,7^ In general, greater parental monitoring has been associated with less total screen time in children^8^ and adolescents.^9^

Given the evolving landscape of digital technology, it is important to investigate how parenting approaches specific to technology use (termed ‘media parenting practices’) influence children’s screen use.^10^ It remains unclear whether or not media parenting practices have significant effects on children’s screen time and screen use behaviors as previous studies have had small sample sizes or mixed findings.^10–13^ Of the existing literature on media parenting practices, a majority have been studied in infants^14^ and younger children^6,8,10,15–20^ with fewer studies focusing exclusively on early adolescents, which have revealed that parental regulation or rules of screen use have been associated with less adolescent screen time, or decreased likelihood of exceeding recommended screen time limits.^12,21,22^ However, some existing evidence points to weaker associations between media parenting practices and screen time for those in middle childhood and adolescence compared to those in early childhood.^23,24^ Early adolescence is a developmental period characterized by a desire for greater independence, which can lead to shifting parent-adolescent relationships.^24,25^ Despite these changes, parental figures continue to play crucial roles in adolescent development, as decreased parental monitoring has been associated with alcohol use, binge drinking, and marijuana use.^26^ Given that screen time has been found to increase in adolescence^23^ and smartphone/Internet engagement is thought to disproportionately impact early adolescents,^27^ it is critical to understand how media parenting practices influence screen use behaviors in this age cohort.

One prior study developed a questionnaire to measure specific media parenting practices, including screen time modeling (e.g., parents’ own screen use behaviors), mealtime screen use, bedroom screen use, using screens to control behavior (e.g., offering screen time as a reward for good behavior), monitoring screen time, and limiting screen time.^10^ However, this study was cross-sectional among 62 children (1.5–5 years old) and their parents in Guelph and Wellington County, Ontario, Canada.^10^ Therefore, understanding the prevalence of media parenting practices at a larger scale and for parents of adolescents remains an important gap in the literature.

Beyond screen time, it is of value to assess the associations of parenting practices with problematic screen use, as parental attitudes towards adolescents and screen use have been found to be risk factors for problematic Internet use.^28^ Problematic screen use can be characterized by addiction-like traits such as tolerance (e.g., feeling the need to use more and more), relapse (e.g., trying to reduce use but unable to), mood modification (e.g., use to forget about problems), salience (e.g., spending a lot of time thinking about use), and conflict (e.g., use has had a bad effect on schoolwork or job), which may disrupt daily functioning.^29–31^ There have been mixed findings regarding the association between parenting practices and problematic screen use in children thus far. One cross-sectional study from the Netherlands suggested that positive parenting and Internet-specific parental rules were associated with fewer problematic social media use behaviors in adolescents.^7^ In contrast, a meta-analysis revealed weak negative associations between problematic Internet use and general parenting practices, such as authoritative parenting, warmth, and control.^32^ Four general parenting styles have been defined in the literature, namely authoritative, authoritarian, indulgent/permissive, and neglectful/uninvolved parenting. Authoritative parenting is characterized by high control and high receptiveness, while authoritarian parenting is defined by high control and low receptiveness. Indulgent/permissive parenting refers to low control and high receptiveness, whereas neglectful/uninvolved parenting is marked by low control and low receptiveness.^8,20,32^ In the same meta-analysis, problematic Internet use was not significantly associated with media-specific parenting practices such as restrictive mediation and active mediation.^32^ Restrictive mediation refers to parental enforcement of media use rules, such as time or content allowed, while active mediation refers to parent-child communication regarding media use.^24,32^ These inconsistencies underscore the need for further studies to investigate effective media-parenting practices that are associated with problematic screen use.

With these gaps in the literature, our study aims to assess the prevalence of various media parenting practices (e.g., screen time modeling, mealtime screen use, bedroom screen use, screens to control behavior, monitoring screen time, limiting screen time) in a diverse national sample of early adolescents in the US. Second, we assess the associations between media parenting practices and adolescent screen time and problematic screen use across social media, video games, and mobile phones.

## Methods

### Study population

The data was collected from the Adolescent Brain Cognitive Development (ABCD) Study, a prospective cohort study which collects annual data on the health and cognitive development of 11,875 adolescents from 21 different geographically diverse sites across the US. The ABCD Study baseline was 2016–2018, when participants were 9-10 years old. The data analyzed in this specific analysis are from the ABCD 5.0 release, which includes the Year 3 follow-up (2019–2022), with 10,048 having media parenting practices and any screen use (screen time or problematic screen use) data to be included in this analysis. Appendix A illustrates the differences in the sociodemographic characteristics of those who were included in any part of the analysis (*N* = 10,048) and those excluded due to missing outcome data (*N* = 1827). This study received centralized institutional review board (IRB) approval from the University of California, San Diego, and the participating study sites received local IRB approval. Written informed consent was provided by caregivers, and written assent was provided by each participating adolescent.

### Media parenting practices

To assess media parenting practices, parents of participating children were asked about their screen time practices through a self-reported questionnaire.^10^ Parents were asked 14 questions (as detailed in Table 2) which were grouped into 6 categories: screen time modeling (measuring parents’ own screen use in front of the adolescent, 2 questions), mealtime screen use (measuring screen use of the entire family during meals, 2 questions), bedroom screen use (measuring adolescents’ screen use in the bedroom, 3 questions), parental control of screen use (measuring the parental control of adolescent screen time for rewards or punishments, 2 questions), parental monitoring of screen use (measuring parental monitoring of the adolescent’s screen use, 2 questions), and parental limiting of screen use (measuring parental limit setting of the adolescent’s screen use, 3 questions). Parents responded to each question based on a 4-point Likert scale with responses ranging from (1) “Strongly Disagree” to (4) “Strongly Agree.” Within the screen time modeling category, the question “I try to limit how much I use a screen-based device when I am with my child” was reverse coded to maintain consistency in the directionality of the responses within the category in the regression model. These scores were then summed and averaged to create average sum scores for each of the 6 categories as has been done previously.^10^

### Adolescent screen use

#### Screen time

Total recreational screen time was calculated using adolescents’ self-reported hours of typical weekday and weekend use of the following: single- and multi-player gaming, texting, social media, browsing the internet, video chatting and watching/streaming movies, videos, or TV.^31,33^ Daily screen time (hours/day) was calculated as a weighted sum [(weekday average x 5) + (weekend average x 2)]/7.^34^ Participants were specifically asked only to include recreational screen use and not to include screen use for school or homework.

### Problematic screen use

#### Problematic social media use (SMAQ)

Adolescents who stated they had at least one social media account (*n* = 6916) were asked to complete the Social Media Addiction Questionnaire (SMAQ),^31^ to assess problematic social media use. The SMAQ consisted of six questions that capture aspects of problematic use such as mood modification (“I use social media apps so I can forget about my problems”), salience (“I spend a lot of time thinking about social media apps or planning my use of social media apps”), relapse (“I’ve tried to use my social media apps less but I can’t”), conflict (“I use social media apps so much that it has had a bad effect on my schoolwork or job”), and tolerance (“I feel the need to use social media apps more and more”). The single-factor model of the SMAQ demonstrated adequate fit in a confirmatory factor analysis (comparative fit index [CFI] = 0.989, root mean square error of approximation [RMSEA] = 0.05 (90% CI: 0.042,0.058)).^31^ In the current sample, the SMAQ demonstrated good internal consistency reliability (α = 0.89).

#### Problematic video game use (VGAQ)

Those who reported any video game use (*n* = 8487) were asked to complete the Video Game Addiction Questionnaire (VGAQ), a self-reported six-question questionnaire modeled after the Bergen Facebook Addiction Scale.^29^ This scale has been used in broader applications to measure video game and social media addiction among adolescents.^30,35^ Questions asked in the VGAQ are similar to the questions described above for the SMAQ to measure mood modification, salience, relapse, conflict, and tolerance, but refer to video games instead of social media. The single-factor model of the VGAQ demonstrated good internal consistency reliability (McDonald’s 0.90) and adequate fit in a confirmatory factor analysis (comparative fit index [CFI] = 0.988, root mean square error of approximation [RMSEA] = 0.060 (90% CI: 0.053,0.067)).^31^ In the current sample, the VMAQ demonstrated good internal consistency reliability (α = 0.86).

#### Problematic mobile phone use

Those who reported using a mobile phone (*n* = 8310), were asked to complete the Mobile Phone Involvement Questionnaire (MPIQ), which consists of eight questions developed to measure elements of behavioral addictions such as conflict, relapse, withdrawal, tolerance, and salience.^36^ Example questions are as follows: “I interrupt whatever else I am doing when I am contacted on my phone” and “I often use my phone for no particular reason”. The responses were based on a 7-point Likert scale ranging from “Strongly Disagree” to “Strongly Agree”.^31^ The questionnaire was developed based on a principal components analysis finding that the eight items included assessed a unitary construct.^36^ In the current sample, the MPIQ demonstrated good internal consistency reliability (α = 0.88).

For each problematic use scale, responses were summed to obtain a total score.

### Statistical analyses

The analyses were performed in Stata 18.0 (StataCorp, College Station, TX). Descriptive statistics, including percentages, means, and SDs were calculated. Multiple linear regression analyses were conducted to estimate associations between exposure variables (six media parenting practices) and outcome variables (total screen time and the three problematic screen use scores). Sociodemographic characteristics, including age, sex, race/ethnicity, parent education, household income, study site, and data collection period (pre- versus during the COVID-19 pandemic using March 13, 2020, as the start date of the pandemic in the US) were considered as covariates in the regression models. Analyses incorporated ABCD propensity scores to approximate the distribution per the American Community Survey from the U.S. Census.^4^

## Results

Table 1 shows the sociodemographic characteristics of the 10,048 individuals included in this analysis. The sample was 48.3% female and included 45.6% from racial/ethnic minority groups. Table 2 shows the frequencies of parent practices around screen use. When asked about screen-time modeling habits, 72.9% of parents stated that they used screens around their adolescents, while 85.3% tried to limit their own screen use when with their adolescents. Over a third (35.6%) of families reported often watching a screen during meals and nearly half (46.2%) of children have access to a mobile screen-based device in bed. Over two-thirds (67.4%) of parents monitor their adolescent’s screen time during the week and three-quarters (76.2%) limit their adolescent’s screen time during the week.

**Table 1:** Sociodemographic and youth screen time characteristics of Adolescent Brain Cognitive Development (ABCD) Study participants at Year 3 (*N* = 10,048).

Sociodemographic characteristics	Mean (SD) / %
Age (years)	12.47 (0.77)
Sex (%)	
Female	48.3%
Male	51.7%
Race/ethnicity (%)	
Asian	5.5%
Black	15.6%
Latino	19.9%
Native American	3.1%
White	54.4%
Other	1.5%
Household income (%)	
Less than $25,000	16.2%
More than $25,000 and less than $50,000	19.9%
More than $50,000 and less than $75,000	17.9%
More than $75,000 and less than $100,000	14.0%
More than $100,000 and less than $200,000	24.2%
Equal to and greater than $200,000	7.7%
Parents’ highest education (%)	
Less than high school education	17.8%
College or more	82.2%
Media parenting practices	
Parental screen time modeling (mean score)	3.05 (0.63)
Mealtime screen use (mean score)	1.86 (0.94)
Bedroom screen use (mean score)	1.82 (0.89)
Use of screens to control behavior (mean score)	2.54 (0.89)
Parental monitoring of screen time (mean score)	2.75 (0.95)
Limiting screen time (mean score)	3.14 (0.72)
Adolescent screen use	
Total screen time (hours/day)	8.67 (8.50)
Problematic social media use (total scores)^a^	8.3 (7.50)
Problematic video game use (total scores)^b^	10.83 (7.67)
Problematic mobile phone use (total scores)^c^	21.39 (13.07)

ABCD Study propensity weights were applied based on the American Community Survey from the US Census.

^a^Asked among a subset who reported social media use (*N* = 6916).

^b^Asked among a subset who reported video game use (*N* = 8487).

^c^Asked among a subset who reported mobile phone use (*N* = 8310).

**Table 2:** Media parenting practices in the Adolescent Brain Cognitive Development (ABCD) Study (*N* = 10,048).

Media parenting practice category and individual questions	Parent responses			
	%	%	%	%
	Strongly disagree	Somewhat disagree	Somewhat agree	Strongly agree
Parental screen time modeling				
“When I am with my child, I use a screen-based device”	11.2%	15.9%	51.3%	21.6%
“I try to limit how much I use a screen-based device when I am with my child”	6.6%	8.1%	40.1%	45.2%
Mealtime screen use				
“Our family often watches a screen during meals”	45.2%	19.2%	23.0%	12.6%
“Family members are allowed to use screen-based devices during meals”	55.5%	19.0%	17.6%	7.9%
Bedroom screen use				
“My child falls asleep while using a screen-based device”	61.5%	14.9%	16.7%	6.9%
“A screen-based device is usually playing in the room when my child falls asleep”	63.5%	11.9%	16.3%	8.3%
“My child has access to a mobile screen-based device in bed”	43.2%	10.6%	25.6%	20.6%
Use of screens to control behavior				
“I offer screen time to my child as a reward for good behavior”	45.6%	15.6%	27.5%	11.3%
“I take away screen time from my child as a punishment for bad behavior”	15.0%	7.1%	30.3%	47.6%
Parental monitoring of screen time				
“I keep track of my child’s screen time during the week”	14.6%	18.0%	38.0%	29.4%
“I keep track of my child’s screen time during the weekend”	16.9%	22.8%	36.5%	23.8%
Limiting screen time				
“I limit my child’s screen time during the week”	11.0%	12.8%	38.3%	37.9%
“I limit my child’s screen time during the weekend”	16.1%	24.9%	36.8%	22.2%
“I encourage my child to do activities other than screen time”	2.9%	1.6%	15.3%	80.1%

ABCD Study propensity weights were applied based on the American Community Survey from the US Census.

Table 3 shows the association between media parenting practices and adolescent-reported screen time and problematic use of social media, video games, and mobile phones adjusting for sociodemographic characteristics. Greater parental screen time modeling (e.g., parents’ own use of a screen-based device when with child) was significantly associated with higher adolescent screen time (B: 0.66, 95% CI: 0.36, 0.96), problematic social media use scores (B: 0.82, 95% CI: 0.55, 1.10), problematic video game use scores (B; 0.38, 95% CI: 0.12, 0.64) and problematic mobile phone use scores (B: 1.31, 95% CI: 0.82, 1.81). Family mealtime screen use was associated with higher adolescent screen time (B: 1.24, 95% CI: 1.01, 1.47) and problematic social media (B: 0.83, 95% CI: 0.64, 1.02), video game (B: 0.53, 95% CI: 0.35, 0.71), and mobile phone (B: 1.33, 95% CI: 1.01, 1.65) use. Adolescents’ bedroom screen use was associated with higher adolescent screen time (B: 1.60, 95% CI: 1.36, 1.85) and problematic social media (B: 1.91, 95% CI: 1.71, 2.11), video game (B: 0.52, 95% CI: 0.32, 0.72), and mobile phone (B: 2.91, 95% CI: 2.56, 3.26) use. Parental control of adolescents’ screen use behavior (e.g., offering screen time as a reward for good behavior) was associated with higher adolescent screen time (B: 0.36, 95% CI: 0.12, 0.60) and higher problematic video game scores (B: 0.64, 95% CI: 0.46, 0.82). Parental monitoring of adolescent screen use was associated with lower adolescent screen time (B: –0.83, 95% CI: –1.05, –0.60), and lower problematic social media (B: –0.86, 95% CI: –1.04, –0.68) and mobile phone use (B: –1.12, 95% CI: –1.44, –0.81) scores. Additionally, parental limiting of adolescent screen use was associated with lower adolescent screen time (B: –1.29, 95% CI: –1.59, –0.98) and problematic social media (B: –1.58, 95% CI: –1.83, –1.34), video game (B: –0.49, 95% CI: –0.72, –0.25), and mobile phone (B: –2.24, 95% CI: –2.66, –1.81) use.

**Table 3:** Associations between media parenting practices and adolescent screen use in the Adolescent Brain Cognitive Development (ABCD) Study (*N* = 10,048).

Media parenting practice categories	Total screen time		Problematic screen use scores					
			Social media		Video game		Mobile phone	
	Coefficient (95% CI)	*p*	Coefficient (95% CI)	*p*	Coefficient (95% CI)	*p*	Coefficient (95% CI)	*p*
Parental screen time modeling	0.66 (0.36, 0.96)	**<0.001**	0.82 (0.55, 1.10)	**<0.001**	0.38 (0.12, 0.64)	**0.004**	1.31 (0.82, 1.81)	**<0.001**
Mealtime screen use	1.24 (1.01, 1.47)	**<0.001**	0.83 (0.64, 1.02)	**<0.001**	0.53 (0.35, 0.71)	**<0.001**	1.33 (1.01, 1.65)	**<0.001**
Bedroom screen use	1.60 (1.36, 1.85)	**<0.001**	1.91 (1.71, 2.11)	**<0.001**	0.52 (0.32, 0.72)	**<0.001**	2.91 (2.56, 3.26)	**<0.001**
Use of screens to control behavior	0.36 (0.12, 0.60)	**0.004**	0.07 (−0.13, 0.27)	0.489	0.64 (0.46, 0.82)	**<0.001**	−0.12 (−0.47, 0.22)	0.488
Parental monitoring of screen time	−0.83 (−1.05, −0.60)	**<0.001**	−0.86 (−1.04, −0.68)	**<0.001**	−0.17 (−0.34, .003)	0.054	−1.12 (−1.44, −0.81)	**<0.001**
Limiting screen time	−1.29 (−1.59, −0.98)	**<0.001**	−1.58 (−1.83, −1.34)	**<0.001**	−0.49 (−0.72, −0.25)	**<0.001**	−2.24 (−2.66, −1.81)	**<0.001**

Coefficient from linear regression model. Models represent the abbreviated output from 24 separate linear regression models including covariate adjustment for age, sex, race/ethnicity, parent education, household income, study site, and data collection period (pre- versus during the COVID-19 pandemic). ABCD propensity weights were applied based on the American Community Survey from the US Census. Bold indicates *p* < 0.05.

## Discussion

In this demographically diverse sample of 12–13-year-old early adolescents in the United States, we found that although 76.2% of parents reported that they try to limit their adolescents’ screen use during the week and 85.3% agreed that they try to limit their own screen use in front of their adolescents, 72.9% of parents report using screens around their adolescents. Approximately one-third of parents allow for family mealtime screen use and adolescent bedtime screen use. Parental monitoring and limiting of adolescent screen use was generally associated with lower adolescent screen time and problematic screen use; however, parental modeling of their own screen use and allowance of mealtime and bedtime screen use was associated with higher adolescent screen time and problematic screen use. Greater parental control of adolescent screen use as a reward or punishment was also associated with higher total screen time and problematic screen use of video games.

### Parental screen time modeling

Our study showed that parental screen use when with their child was associated with higher total screen time and problematic social media, video games, and mobile phone use in early adolescents. These findings were consistent with various prior studies, which have suggested that greater parental screen time use is associated with greater screen time in younger children^17,18,20,37^ and more frequent co-use of screens with children.^13^ These associations could potentially be explained in the context of social learning theory, which states that individuals learn from observing and modeling other’s behavior. Children may mirror parental behavior and could thus model their parents’ screen use behaviors.^7^ It is also possible that parents who use digital media more frequently may be more open to children’s media use and impose fewer restrictions.^13^

### Mealtime & bedroom screen use

Family mealtime screen use and child bedroom screen use were both positively associated with adolescent total screen time and problematic use of social media, video game, and mobile phone, which is in accordance with prior evidence.^6,10,11,15,38^ The American Academy of Pediatrics advocates for a Family Media Use Plan which notes that parents may consider instituting screen-free times such as during mealtime and bedtime. Watching screens during meals has been linked to overeating, distracted eating, and weight gain/obesity.^3,39^ Bedtime screen use has been linked with shorter sleep duration and sleep disturbances, potentially due to higher arousal at bedtime, blue light effects, and disturbances by notifications.^5^ One prior study during the COVID-19 pandemic found no association between the implementation of parent rules (including limiting screen use at mealtimes and bedtime) and problematic media use; however, the analyses grouped all parent media rules together and did not differentiate between mealtime and bedtime screen rules.^40^ To our knowledge, prior studies have not examined whether parental limiting of bedtime and mealtime screen use reduces adolescent total screen time and problematic screen use; further research may shed light on this topic.

### Use of screens to control behavior

Interestingly, in our study, while a little over three-quarters of parents reported removing screen time as a punishment for bad behavior, almost 40% reported offering screen time as a reward for good behavior. Greater control of adolescent screen use as a reward or punishment was associated with increased total screen time and problematic screen use of video games. Our findings support prior studies, which revealed that screen-based devices as disciplinary tools increased children’s screen time.^10,41^ However, our findings contrast with another study that did not find an association between rewarding screen time and children’s weekly TV viewing.^15^ In our study, no significant associations were found between screens to control behavior and problematic social media and mobile phone use. One potential explanation is that early adolescents are at a stage of seeking independence away from their parents/caregivers and may view certain media parenting practices as intrusive, leading them to reject rules.^13,17,23,25,42^ With the increasing accessibility, availability, and familiarity of digital media platforms, early adolescents may increasingly depend on these modalities for social support and identity exploration.^43^

Parenting style may be of consideration in this context. One European randomized controlled trial on 10–12-year-old children revealed that autonomy-supportive parenting styles were associated with less TV/DVD and computer/game console time while controlling parental styles were associated with perceived excessive time on TV/DVD and computer/game consoles.^21^ One cross-sectional study from the Netherlands found that more problematic internet use was associated with less positive parenting practices such as rejection and harsh punishment.^7^ However, these more negative parenting practices may reflect general parenting styles rather than ones specific to screen use.

### Parental monitoring of screen time and limiting screen time (parental restriction of screen use)

Parental monitoring of screen time was inversely associated with total screen time and problematic social media and mobile phone use, which parallels prior findings that greater parental screen time monitoring was associated with lower children’s screen time^10,15^ and fewer problematic social media use behaviors^7^ respectively. Parental monitoring of screen time tended to be inversely associated with problematic video game use; however, the association was not statistically significant. In our questionnaire, parental monitoring of screen use does not necessarily involve actions to limit or control screen use, which are measured separately. Further research may be needed to determine how parental control of adolescent screen use as a reward or punishment can differ from monitoring screen time in affecting problematic video game use. Parental limiting of screen time was also inversely associated with total screen time and problematic social media, video games, and mobile phone use in early adolescents. This is in accordance with prior evidence, which has found that family screen time rules, including limiting screen time, are associated with children spending less time watching TV/DVD and using the computer or game console.^15,17,21^ Setting screen time boundaries is in line with characteristics of authoritative parenting, in which parents impose control and are responsive and supportive of their children, a parenting style that has been associated with positive developmental results such as healthy dietary behavior and improved academic outcomes.^8,20^

### Limitations and strengths

There are several limitations of our study to be considered. With the cross-sectional nature of the study, directions of causality cannot be determined. Though potential confounders were adjusted for, it is possible that there are remaining confounders. There is the possibility of selection bias as participants from racial/ethnic minority populations and lower socioeconomic backgrounds were less likely to be included in the analytic sample. All measures were self-reported, which increases the possibility of reporting and recall bias. The Video Game Addiction Questionnaire was based on a measure developed for social media, although it similarly captures elements of behavioral addictions such as mood modification, salience, relapse, conflict, and tolerance.^29,31^ Our screen use measures were not able to differentiate between active (e.g., active or interactive engagement with cognitive, physical, or social tasks) versus passive (e.g., passive absorption of information, such as watching media) screen use, which could be an area of future research. The screen time measure focused on recreational screen time rather than screen time related to school or school work, which could be investigated in relation to parent rules and monitoring in future research. Our bedroom screen use measure captured if children had access to a mobile screen-based device in the bedroom, but did not gather information about specific bedroom screen use rules. This may be a future area to investigate, as the absence of parental bedtime screen rules has been found to exacerbate social media frequency effects in adolescents.^44^

It is also important to consider that the data in this study include Year 3 follow-up data from 2019 to 2022, overlapping with the years of the COVID-19 pandemic which has had a profound impact on screen use. On average, screen time in children and adolescents was found to have increased by 52% during the pandemic.^45^ Adolescents between the ages of 12 and 18 had greater changes in screen time when compared to their younger counterparts and were more likely to have access to personal devices. With the social distancing restrictions in place during the pandemic, children and adolescents were less likely to engage in in-person social interactions, thus likely turning to digital media to maintain their social networks. Therefore, it should be noted that our findings may be influenced by the effects of increased screen time and shifting individual and family dynamics from the COVID-19 pandemic during this period of isolation.

Strengths of this study include its large, diverse sample of early adolescents in the United States and measures of total screen time and problematic screen use based on adolescent reports. The present study advances prior work by encompassing adolescent screen use behaviors of contemporary digital modalities and analyzing problematic screen use per modality, rather than collapsing devices into one measure.

## Conclusion

With the potential risks of children’s excessive screen time and problematic screen use in relation to negative health outcomes, there are several implications from the current study. The American Academy of Pediatrics encourages the creation of Family Media Use plans.^46^ Our findings suggest that monitoring and limiting screen time parent media practices are associated with lower screen time and lower problematic screen use in early adolescents. In contrast, using screens to control behavior (i.e., as a reward or punishment) is associated with greater total screen time and problematic screen use of video games. These actions could be incorporated into a family media use plan, as parents may have discussions with their children regarding setting boundaries on screen time, while minimizing use of screens to control behavior as a reward or punishment, to prevent downstream screen use effects. The implementation of a family media use plan may be more successful when clear, consistent rules are mutually agreed upon by parents and children.^10,11,19,22^ This is particularly important for early adolescents who may spend longer portions of the day away from home and are developing more autonomy. Recreational screen use is generally higher on weekends than on weekdays; future research may investigate the differences in parenting media practices on weekdays compared to weekends, and associations with adolescent screen use.^47^ Future research may explore more objective measures of screen use behaviors to compare the efficacy of various media parenting practices to further shape public health policy and clinical guidance for early adolescents.

## Supplementary information

Appendix A R1

## Data Availability

Data used in the preparation of this article were obtained from the ABCD Study (https://abcdstudy.org), held in the NIMH Data Archive (NDA). Investigators can apply for data access through the NDA (https://nda.nih.gov/).
