# Supplementary material for: Associations between media parenting practices and early adolescent screen use
Source: Pediatr Res. 2024 Jun 5;97(1):403–10. doi: 10.1038/s41390-024-03243-y (PMC11626836; doi:10.1038/s41390-024-03243-y)
Supplement: Supplementary file 1 — Appendix A R1 [file 41390_2024_3243_MOESM1_ESM.pdf]

Appendix A. Comparison of participants included vs excluded from the Adolescent Brain Cognitive Development (ABCD) Study

Sociodemographic characteristics	Included (n=10,048)	Excluded (n=1,827)	p
Sex (%)			0.055
Female	48.3%	51.2%	
Male	51.7%	48.8%	
Race/ethnicity (%)			<0.001
Asian	5.5%	5.4%	
Black	15.6%	27.2%	
Latino	19.9%	20.8%	
Native American	3.1%	3.3%	
White	54.4%	41.7%	
Other	1.4%	1.5%	
Household income (%)			<0.001
Less than $25,000	16.2%	31.8%	
More than $25,000 and less than $50,000	19.9%	21.8%	
More than $50,000 and less than $75,000	17.9%	16.5%	
More than $75,000 and less than $100,000	14.0%	9.6%	
More than $100,000 and less than $200,000	24.2%	14.6%	
Equal to and greater than $200,000	7.7%	5.6%	
Parents' highest education (%)			<0.001
Less than high school education	17.8%	32.2%	
College or more	82.2%	67.8%	

Propensity weights were applied based on the American Community Survey from the US Census.
